# EVALUATION OF EARLY TREATMENT OF IDIOPATHIC CLUBFOOT USING THE PONSETI METHOD

**DOI:** 10.1590/1413-785220233102e259899

**Published:** 2023-05-01

**Authors:** CAIO LUIZ DE TOLEDO OLIVEIRA, GERALDO MOTA GONÇALVES, JOSÉ BATISTA VOLPON

**Affiliations:** 1 Universidade de Sao Paulo, Faculdade de Medicina de Ribeirão Preto, Ribeirão Preto, SP, Brazil.; 2 Universidade de Sao Paulo, Faculdade de Medicina de Ribeirão Preto, Hospital das Clínicas, Departamento de Ortopedia e Anestesiologia, Ribeirão Preto, SP, Brazil.

**Keywords:** Congenital Abnormalities, Foot Deformities, Clubfoot, Malformações Congênitas, Deformidades do Pé, Pé Torto Equinovaro

## Abstract

Congenital clubfoot is one of the most common congenital deformities of the lower limbs, with an estimated incidence of 1 for every 1,000 live births. Its treatment is controversial, and currently the Ponseti method has been widespread, showing promising results and decreasing the need for extensive surgical releases, as was usually done until the introduction of the technique. Currently used in approximately 55 countries, the method has changes and scopes that vary according to the society in which it is applied, with the results and peculiarities of the method molded according to the sample studied.

**Objective:**

To evaluate the clinical outcome of clubfoot treatment using the Ponseti method under local conditions.

**Methods:**

The clinical evaluation will include a descriptive analysis of the sample, as well as radiographic evaluation and family satisfaction with the treatment.

**Results:**

In total, 46% of the patients had good results and no family was dissatisfied with the treatment results. No statistically relevant relationships were found between the studied variables.

**Conclusion:**

The results are good and generally similar to those in the literature. Our epidemiological data generally agree with those reported by other authors. ***Level of Evidence IV, Case Series.***

## RESUMO

O pé torto congênito (PTC) é uma das deformidades congênitas mais comuns dos membros inferiores, com incidência estimada de um para cada 1.000 nascidos vivos. Seu tratamento é motivo de controvérsia, mas, atualmente, o método de Ponseti tem sido difundido, mostrando resultados promissores e diminuindo a necessidade das liberações cirúrgicas extensas, como geralmente se fazia até a introdução da técnica. Utilizado em aproximadamente 55 países, o método apresenta alterações e alcances que variam de acordo com a comunidade em que é aplicado, sendo o resultado e as peculiaridades do método moldados de acordo com a amostra estudada.

**Objetivo:**

Avaliar o resultado clínico do tratamento do PTC através do método de Ponseti em condições locais.

**Métodos:**

A avaliação clínica incluiu uma análise descritiva da amostra, além de avaliação radiográfica e satisfação da família com o tratamento.

**Resultados:**

46% dos pacientes apresentaram bons resultados e nenhuma família se mostrou insatisfeita. Não foram encontradas relações estatisticamente relevantes entre as variáveis estudadas.

**Conclusão:**

Os resultados são bons e, de maneira geral, semelhantes àqueles da literatura. Há, também, concordância geral dos dados epidemiológicos deste estudo com os relatados por outros autores. **Nível de Evidência IV, Série de Casos.**

## INTRODUCTION

Congenital clubfoot (CCF) is a complex three-dimensional deformity that results from the association of equinus, cavus, varus, and adductus foot.^1^ The condition has an approximate incidence of one case per 1,000 live births among white people, but this number varies according to the population studied^2^ and affects both sexes and ethnicities. ^(^ ^3^ Most CCF cases occur isolated, called “idiopathic” (iCCF) due to their unknown cause. However, around 20% of cases are associated with underlying diseases, classified as “teratological.” ^(^ ^4^

The treatment has been challenging, with manipulations and bandages being recommended by Hippocrates. Therapy has become more aggressive, with wrench-like devices being developed, which forced the correction of the foot, actually crushing the bones. ^(^ ^5^ Then, conservative treatment was reestablished, culminating in Kite, ^(^ ^6^ which was slow and presented uncertain results. Kite’s technique was associated with early but increasingly extensive surgical releases until reaching the “circumferential release of the foot”, ^(^ ^7^ still unsatisfactory.

This scenario changed with Laaveg and Ponseti,^8^ who created a new concept of the type of manipulation and plaster cast, associated or not with percutaneous Achilles tenotomy and a prolonged use of the Denis Browne splint. This resulted in well-corrected and flexible feet and lower rate of recurrences.

Due to its effect, this method is now widespread, well established, being used in over 50 countries. ^(^ ^9^ Many Brazilian authors have already published their results, which were mostly favorable. ^(^ ^10^ ^)- (^ ^12^

Our institution has been using this method for several years; however, the results must be always reevaluated and compared to those of the literature. Thus, this study aimed to analyze the results of cases of idiopathic congenital clubfoot treated early by the Ponseti method.

## METHODS

This is a retrospective observational study of data from patients with idiopathic congenital clubfoot, who underwent treatment with the Ponseti method from 2011 to 2016. This study was approved by the Research Ethics Committee of the Hospital das Clínicas of the Ribeirão Preto Medical School of the University of São Paulo (Opinion No. 13,034-2019, approved on 12/02/2019). Individuals who started treatment until three months of age were included, whereas those who started treatment in another institution or were treated by other methods were excluded. Patients with irregular follow-up, who interrupted the treatment, or whose medical records were unavailable were also excluded. The minimum follow-up time after treatment was established as 12 months.

In total, 142 children with congenital clubfoot were treated during this period, 53 of which being included in the study, totaling 74 feet. Data such as gender, laterality, classification according to Pirani, Staheli, and Naddumba, ^(^ ^13^ number of exchanges, need for tenotomy, recurrences, and follow-up time were obtained.

**Figure 1:**
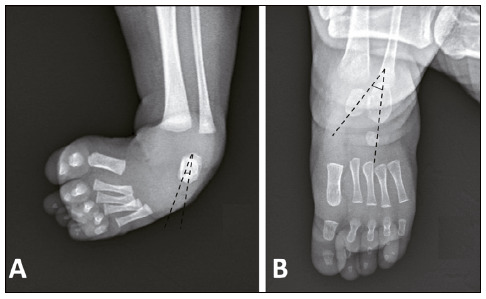
Illustration of the determined longitudinal axes of the talus and calcaneus on radiographs in the anteroposterior incidence of the foot (Kite’s angle). (A) The idiopathic congenital clubfoot presents overlap of the two bones expressed by the smallest opening angle between them; (B) the corrected foot presents separation between the bones and the shafts are very divergent.

Source: Arquivo da Disciplina de Ortopedia Pediátrica e Afecções do Pé, Hospital das Clínicas of the Ribeirão Preto Medical School of the Universidade de São Paulo.

**Figure 2:**
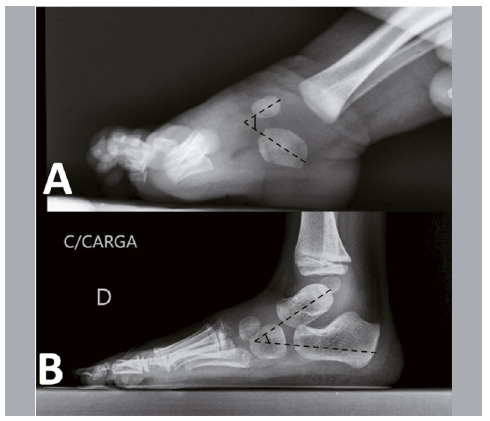
Illustration of the determined angle between the talus and calcaneus in the lateral incidence (Kite’s angle). (A) Before treatment; (B) after treatment. When the foot is not corrected, the angle vertex is in the dorsal region. After correction, the angle should be on the midfoot, preferably on the cuboid ossification center.

Source: Arquivo da Disciplina de Ortopedia Pediátrica e Afecções do Pé, Hospital das Clínicas of the Ribeirão Preto Medical School of the Universidade de São Paulo.

**Figure 3:**
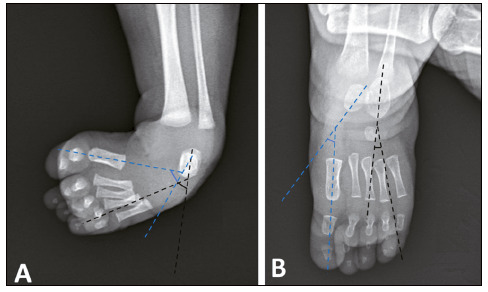
Illustration of the determined longitudinal axes of the talus and calcaneus on radiographs in the anteroposterior incidence of the foot. In the radiographs in the anteroposterior incidence, lines were drawn based on the longitudinal axes of the talus and calcaneus, along the first metatarsal, as well as in the space between the fourth and fifth metatarsals for evaluation of the forefoot deviation. (A) In the idiopathic congenital clubfoot, these lines are broken, and the corresponding angles increased; (B) in the normal foot, the longitudinal axis of the talus coincides or points medially to the bone of the first metatarsal, and the axis of the calcaneus coincides with the alignment between the fourth and fifth metatarsals.

Source: Arquivo da Disciplina de Ortopedia Pediátrica e Afecções do Pé, Hospital das Clínicas of the Ribeirão Preto Medical School of the Universidade de São Paulo.

In the institution, treatment is initiated by foot manipulation, according to Ponseti’s strict orientation, ^(^ ^14^ then, plaster immobilization is performed in the position in which the foot could be manipulated. Radiographic evaluation was performed in the first and last appointments. Kite’s angles (talocalcaneal) were measured in the anteroposterior and profile incidences before and after treatment (Figures 1 and 2), as well as the relation between the forefoot and hindfoot bones using the relation between the long axes of the talus and calcaneus with the first and fourth metatarsals, respectively (Figure 3).

The Assessing Clubfoot Treatment (ACT) satisfaction index^15^ was applied (Chart 1). Information were obtained from the families by phone calls. Out of the 53 cases, 33 families were contacted (44 feet).

**Chart 1:** Questionnaire to evaluate clinical results, according to family members.

**Score**	**Questions**			
	What do you consider to be the shape of your child's foot?	Does your child complain of foot pain?	Can your child wear any type of footwear?	Are you satisfied with the treatment of your child's feet?
	**Answer option**			
**0**	Very altered	Yes, and with frequent limitations	Never	Very dissatisfied
**1**	Altered	Yes, but it rarely limits their activities	Rarely	Dissatisfied
**2**	Almost normal	Yes, but it does not limit their activities	Almost always	Satisfied
**3**	Normal	No	Always	Very satisfied

Simple and multiple logistic regressions were used to test the associations between treatment satisfaction and potential variables. Statistical analyses were performed with the RStudio software (version 1.1.456), and all tests considered p < 0.05 as statistically significant.

## RESULTS

Out of the 53 patients selected for the study, 35 (66%) were boys and 18 (34%) girls; 34 had unilateral iCCF (64%), 18 in the left foot (52%), and 16 in the right foot (48%). Statistical relationship between sex and either good or bad results (p = 0.93), or between bilaterality and treatment results (p = 0.33), was not found. Mean follow-up time was 50 months, with a minimum of 12 months, and a maximum of 98 months. Mean number of plaster exchanges was 7.41 per patient, with a minimum of four and maximum of 16. Mean age at the beginning of treatment was 42 days, with a minimum of four days and maximum of 82 days. Likewise, no statistically relevant relationship was found between the number of exchanges and treatment results (p = 0.11).

Out of the 74 feet evaluated, 63 (85%) underwent percutaneous Achilles tenotomy. Mean initial Pirani score was 5.1, suggesting the occurrence of severe feet. We found no statistical relationship between the Pirani classification score and treatment results (p = 0.13).

We observed a mean increase of 18.73° in the talocalcaneal angle, in the anteroposterior incidence after treatment (178%). In total, 38 feet (51.3%) presented a talocalcaneal angle greater than or equal to 30° after treatment, a value considered satisfactory by Ponseti. In the relationship between the hindfoot bones, 21 out of the 71 feet (29.5%) presented correction of the axes after treatment. In the lateral incidence, 23 (44.1%) of the feet showed a reduction in the angle, whereas 28 (54.9%) showed an increase; 23 of them could not be compared.

Out of the 21 recurrences (40%), 18 patients required surgical interventions and, of these, 12 (57%) presented reports of incorrect orthosis use during follow-up in the medical records. Considering the number of feet, 33 out of 74 (39%) presented recurrence, and of these, 26 required surgery (35%). In total, 15 feet were subjected to anterior tibial translation associated or not with combined wedge osteotomy in the cuboid and navicular. Recurrences in four patients were treated exclusively with a plaster cast, and two were subjected to *à la carte* posteromedial release.

All 33 family members contacted (47 feet) answered the questionnaire. Regarding pain, 25 (75.7%) reported no complaints, six (18.1%) reported sporadic pain that did not limit activities. Two cases (6.2%) reported pain complaints with limitation of activities, even if not very often.

Regarding footwear wear, 23 families (69.6%) answered that their child wore any type of footwear, and two patients were could rarely wear any footwear (6%).

Regarding foot shape, 10 families (30.3%) answered that it was normal, 15 families (45.5%) reported that the feet were practically normal, seven families (21.2%) reported that the children had feet different than the usual, and one family (3%) reported that one foot presented a very different shape.

Regarding general satisfaction with the treatment, 22 families (66.6%) were very satisfied, 11 families (33.4%) were satisfied, and no family declared to be dissatisfied or very dissatisfied with the treatment. Table 1 shows data and clinical score.

**Table 1:** Degree of satisfaction, score from 0-12 points.

Score	Number of families	Percentage	Results
**6**	1	3%	Bad
**7**	1	3%	
**8**	5	15%	
**9**	5	15%	Regular
**10**	6	18%	
**11**	6	18%	Good
**12**	9	28%	
**Total**	**33**	**100%**	

Seven families scored ≤ 8 (21.2%), whereas 15 (45.5%) scored 11 and 12. Eleven families scored 9 or 10 (33.3%).

Simple and multiple logistic regressions were used to test the associations between treatment satisfaction and potential variables. None of the correlations studied were statistically significant.

## DISCUSSION

The Ponseti method is considered a major advance in the treatment of CCF, with unprecedented results, as many feet achieved a normal or almost normal appearance and maintained flexibility. Recurrences are usually not severe, being corrected with new plasters or smaller surgeries. If surgery is required, anterior tibial translation is the most common. ^(^ ^14^ Overall, our results confirm these impressions. The epidemiological data of our population were similar to those reported in the literature regarding gender, ^(^ ^2^ bilaterality, ^(^ ^2^ ^), (^ ^16^ slightly differing regarding the similar involvement of the sides when compared with other reports, ^(^ ^2^ ^), (^ ^16^ ^), (^ ^17^ which probably represents only regional and typical variations of the sample. In Brazilian literature, Jaqueto et al. ^(^ ^12^ found that 64.5% of males were affected and 80.6% of incidence in the right foot.

Our results of mean plaster exchange before indicating tenotomy are also in accordance with most of the reports. ^(^ ^18^ ^), (^ ^19^ This information is important as it reflects the proper indication of the technique and the adherence of the families. When the number of plaster exchanges is very discrepant, they serve as a warning for the identification of possible problems, especially for public institutions working with resident physicians. Although Lourenço and Morcuende^20^ reported that 90% of their patients required less than five plaster changes; this result may reflect local characteristics not observed in other regions or countries.

Regarding the need for Achilles tenotomy for equine correction, this occurred in 85% of our sample, similarly to that of other authors. ^(^ ^14^ ^), (^ ^18^ ^), (^ ^20^

The percentage of recurrences is a very relevant data in the treatment of any deformity and in this case was around 40%. In his first series, Ponseti showed that up to 56% of his patients also needed a new approach after the end of treatment, whether surgery or plaster exchange. ^(^ ^14^ However, “recurrence” is comprehensive term that can be subjective. We evaluated the association between recurrences and the number of plaster changes (p = 0.96), initial Pirani score (p = 1.00), need for tenotomy, and age at the beginning of treatment (p = 0.72), and found no statistically significant association. The concepts of these parameters are also not clear in the literature, which makes difficult a comparison against other results.

Originally, radiographic measurements had been proposed by Ponseti since his first publications on the method; however, good correlations between radiographic findings and clinical findings could never be established. Our analysis showed that 51.3% of the feet had a talocalcaneal angle above 30°. Considering only radiographic parameters, the correction rate in our patients would be 29.5-51%. In the lateral incidence, Kite’s angle measurement presents great variation in normal patients, with normal considered 17-46°.^14^ Among the radiographs analyzed, only 28 (54%) of them presented an increase in the talocalcaneal angle that would be compatible with a decrease in the varus of the hindfoot. These results reinforce Ponseti’s statement that the cases present clinicoradiographic dissociation. Other authors have also shown the limitation of radiographs to evaluate the Ponseti method, as they seem not that much useful to evaluate the efficacy of treatment if analyzed separately. ^(^ ^21^

Ponseti also proposed a system for evaluating CCF results that considers six parameters, in which the sum of their scores would have a maximum of 100 points. ^(^ ^14^ Recently, Smythe et al. ^(^ ^15^ presented a tool called ACT (Assessing Clubfoot Treatment) to establish a score that shows a good relationship with clinical evaluation. Given the many different forms of evaluations of CCF treatment, none of them seem to be validated and full accepted as standard. The results of our functional clinical evaluation showed that five patients (15.1% of those contacted) scored ≤ 8, which would require a revaluation; however, no family reported dissatisfaction with the results of the treatment. This scenario may indicate the low expectation of family members regarding the result, especially when compared with the initial aspect of the foot.

This study has limitations for data analysis and association due to the observational retrospective design. Studies on CCF must carefully evaluate sample profile, treatment results and its complications. Further studies can be conducted implementing follow-up protocols that facilitate data collection and standardization, as well as objective and functional resources to access the results, such as baropodometry and gait analysis, which, however, could only be addressed in studies of a prospective design. Furthermore, evaluation of flexibility and muscle strength of the feet should have been included, which were disregarded since we found no objective methods in the literature that could be applied in children of different ages.

In short, the comparison of our results mostly agrees with those of the literature, presenting epidemiological data within parameters already known, weak association between radiological and clinical results, and 46% of good results with treatment.

## CONCLUSION

The results of iCCF treatment by the Ponseti method in the population studied are good and generally similar to those in the literature. Our epidemiological data also generally agrees with those reported by other authors.

## References

[1] Wynne-Davies R (1964). Family studies and the cause of congenital club foot. Talipes equinovarus, talipes calcaneo-valgus and metatarsus varus. J Bone Joint Surg Br.

[2] Chung CS, Nemechek RW, Larsen IJ, Ching GHS (1969). Genetic and epidemiological studies of clubfoot in Hawaii: general and medical considerations. Hum Hered.

[3] Penny JN (2005). The neglected clubfoot. Tech Orthop.

[4] Wynne-Davis R (1972). Genetic and environmental factors in the etiology of talipes equinovarus. Clin Orthop Relat Res.

[5] Peltier LF (1993). Orthopedics: a history and iconography.

[6] Kite JH (1939). Principles involved in the treatment of congenital club-foot. J Bone Joint Surg Am.

[7] McKay DW (1983). New concept of and approach to clubfoot treatment: section II - correction of the clubfoot. J Pediatr Orthop.

[8] Laaveg SJ, Ponseti IV (1980). Long-term results of treatment of congenital club foot. J Bone Joint Surg Am.

[9] Owen RM, Capper B, Lavy C (2018). Clubfoot treatment in 2015: a global perspective. BMJ Glob Health.

[10] Lara LCR, Montesi DJC, Prado FR, Barreto AP (2013). Tratamento do pé torto congênito idiopático pelo método de Ponseti: 10 anos de experiência. Rev Bras Ortop.

[11] Chueire AJFG, Carvalho G, Kobayashi OY, Carrenho L (2016). Tratamento do pé torto congênito pelo método de Ponseti. Rev Bras Ortop.

[12] Jaqueto PA, Martins GS, Mennucci FS, Bittar CK, Zabeu JLA (2016). Functional and clinical results achieved in congenital clubfoot patients treated by Ponseti&apos;s technique. Rev Bras Ortop.

[13] Pirani S, Staheli L, Naddumba E (2008). Ponseti clubfoot management: teaching manual for healthcare providers in Uganda.

[14] Ponseti IV (1996). Congenital clubfoot: fundamentals of treatment.

[15] Smythe T, Mudariki D, Gova M, Foster A, Lavy C (2019). Evaluation of a simple tool to assess the results of Ponseti treatment for use by clubfoot therapists: a diagnostic accuracy study. J Foot Ankle Res.

[16] Barker SL, Macnicol MF (2002). Seasonal distribution of idiopathic congenital talipes equinovarus in Scotland. J Pediatr Orthop B.

[17] Werler MM, Yazdy MM, Mitchell AA, Meyer RE, Druschel CM, Anderka M (2013). Descriptive epidemiology of idiopathic clubfoot. Am J Med Genet A.

[18] Abdelgawad AA, Lehman WB, van Bosse HJP, Scher DM, Sala DA (2007). Treatment of idiopathic clubfoot using the Ponseti method: minimum 2-year follow-up. J Pediatr Orthop B.

[19] Bor N, Coplan JA, Herzenberg JE (2009). Ponseti treatment for idiopathic clubfoot: minimum 5-year followup. Clin Orthop Relat Res.

[20] Lourenço AF, Morcuende JA (2007). Correction of neglected idiopathic club foot by the Ponseti method. J Bone Joint Surg Br.

[21] Simons GW (1978). A standardized method for the radiographic evaluation of clubfeet. Clin Orthop Relat Res.

